# Magnetic resonance imaging indicators of blood-brain barrier and brain water changes in young rats with kaolin-induced hydrocephalus

**DOI:** 10.1186/2045-8118-8-22

**Published:** 2011-08-11

**Authors:** Marc R Del Bigio, Ili Slobodian, Angela E Schellenberg, Richard J Buist, Tanya L Kemp-Buors

**Affiliations:** 1 Department of Pathology, University of Manitoba; 401 Brodie Centre, 727 McDermot Avenue, Winnipeg MB R3E 3P5 Canada; 2 Manitoba Institute of Child Health; 715 McDermot Avenue, Winnipeg, MB R3E 3P4 Canada; 3 formerly Department of Pharmacology and Therapeutics, University of Manitoba; 753 McDermot Avenue, Winnipeg MB, R3E 0T6 Canada; current address Department of Surgery, University of Saskatchewan; 103 Hospital Drive, Suite 2646, Saskatoon SK, S7N 0W8 Canada; 4 Department of Radiology, University of Manitoba; A006 Chown Building, 753 McDermot Avenue, Winnipeg MB R3E 0W3 Canada; 5 formerly Manitoba Institute of Child Health; current address Department of Paediatrics, University of Calgary; 2888 Shaganappi Trail NW, Calgary AB, T3B 6A8 Canada

**Keywords:** Hydrocephalus, tissue water, density, magnetic resonance, endothelium

## Abstract

**Background:**

Hydrocephalus is associated with enlargement of cerebral ventricles. We hypothesized that magnetic resonance (MR) imaging parameters known to be influenced by tissue water content would change in parallel with ventricle size in young rats and that changes in blood-brain barrier (BBB) permeability would be detected.

**Methods:**

Hydrocephalus was induced by injection of kaolin into the cisterna magna of 4-week-old rats, which were studied 1 or 3 weeks later. MR was used to measure longitudinal and transverse relaxation times (T1 and T2) and apparent diffusion coefficients in several regions. Brain tissue water content was measured by the wet-dry weight method, and tissue density was measured in Percoll gradient columns. BBB permeability was measured by quantitative imaging of changes on T1-weighted images following injection of gadolinium diethylenetriamine penta-acetate (Gd-DTPA) tracer and microscopically by detection of fluorescent dextran conjugates.

**Results:**

In nonhydrocephalic rats, water content decreased progressively from age 3 to 7 weeks. T1 and T2 and apparent diffusion coefficients did not exhibit parallel changes and there was no evidence of BBB permeability to tracers. The cerebral ventricles enlarged progressively in the weeks following kaolin injection. In hydrocephalic rats, the dorsal cortex was more dense and the white matter less so, indicating that the increased water content was largely confined to white matter. Hydrocephalus was associated with transient elevation of T1 in gray and white matter and persistent elevation of T2 in white matter. Changes in the apparent diffusion coefficients were significant only in white matter. Ventricle size correlated significantly with dorsal water content, T1, T2, and apparent diffusion coefficients. MR imaging showed evidence of Gd-DTPA leakage in periventricular tissue foci but not diffusely. These correlated with microscopic leak of larger dextran tracers.

**Conclusions:**

MR characteristics cannot be used as direct surrogates for water content in the immature rat model of hydrocephalus, probably because they are also influenced by other changes in tissue composition that occur during brain maturation. There is no evidence for widespread persistent opening of BBB as a consequence of hydrocephalus in young rats. However, increase in focal BBB permeability suggests that periventricular blood vessels may be disrupted.

## Background

Hydrocephalus is defined as an abnormal intracranial accumulation of cerebrospinal fluid (CSF), usually within the cerebral ventricles. It has been inferred from computed tomography (CT), magnetic resonance (MR), tissue density (specific gravity) measurements, and electrical impedance studies in humans and experimental animals that compression of brain gray matter occurs at the expense of tissue water content [1-4]. Furthermore, experimental tracer studies show impaired diffusion through cortical gray matter [5,6] and ultrastructural studies show compression of the extracellular compartment in the outer cortical layers [7,8]. These observations partially support Hakim's hypothesis, which posited that brain tissue is compressed in the hydrocephalic state [9]. However, it is also clear that extracellular water content and volume is increased in the periventricular white matter animals and humans with hydrocephalus [10-12].

Changes in brain water content often occur in concert with blood brain barrier (BBB) alterations [13]. Impairment of BBB integrity in association with hydrocephalus has been suggested, however there are inconsistencies in the literature [14,15]. Using proflavine hydrochloride (3,6-diaminoacridine hydrochloride, MW 246 Da) injected intraperitoneally, vascular permeability was identified only in the circumventricular organs of mutant hydrocephalic mice [16]. Aquaporin 4 null mice, which develop hydrocephalus, have intact BBB as judged by intravenous injection of Evans blue dye (which forms a complex with plasma albumin, MW 69 kDa) [17]. Perfusion of ^125^I-albumin (MW 69 kDa) or type II horseradish peroxidase (HRP; MW ~44 kDa) into the ventricles of cats with kaolin-induced hydrocephalus revealed no reverse flow across the BBB [18,19]. Similarly, in rats with kaolin-induced hydrocephalus, neither HRP nor microperoxidase (MW 1905 Da) injected into the ventricle crossed the endothelium. Lanthanum chloride (MW 245 Da) was able to traverse the endothelial tight junctions, although there were no normal animals for comparison [20]. Electron microscopic study of blood vessels in hydrocephalic rats showed small foci of endothelial separation, but HRP injected intravenously did not cross the blood vessel walls [21]. Similar endothelial changes were reported in brain biopsies from human hydrocephalic patients [22-24]. Immunohistochemical detection of BBB related antigens (including P-glycoprotein) is altered in the white matter capillaries of hydrocephalic H-Tx rat brains at 4 weeks of age [25,26]. Conversely, young rats with kaolin-induced hydrocephalus exhibited no changes in the endothelial transport molecules RAGE (receptor for advanced glycation end) or LRP-1 (low-density lipoprotein receptor-related protein) [27], while hydrocephalus in 12 month rats was associated with diminished LRP-1 immunoreactivity [28]. Using positron emission tomography (PET) imaging, hydrocephalic human infants showed no evidence for increased BBB permeability to ^82^Rb^+^, a positron-emitting analogue of K^+ ^[29]. To summarize, some structural studies suggest changes in endothelia of hydrocephalic brain, tracer studies using large molecules do not show increased permeability of the BBB in either direction, while tracer studies using small molecules has yielded inconsistent results.

Based on the conflicting literature we also decided to pursue further the topic of hydrocephalus and BBB disruption. We hypothesized that hydrocephalus induced by injection of kaolin into the cisterna magna of young rats would lead to blood brain barrier disruption as detected by efflux of small and large lysine-fixable fluorescent dextran tracers [30] into brain parenchyma and by contrast enhanced MR imaging using gadolinium diethylenetriamine penta-acetate (Gd-DTPA; MW 590 Da) [31]. The secondary goal of the current study was to correlate *in vivo *magnetic resonance (MR) parameters presumed to be influenced by tissue water [3] with water content in brain tissue of immature hydrocephalic rats. Our hypothesis was that hydrocephalus-induced changes in brain water would differ depending on the anatomical region studied and that the MR changes would be dictated by the changes in water content [32,33].

## Materials and methods

### Animal preparation

All animals were treated in accordance with the guidelines of the Canadian Council on Animal Care and the institutional animal ethics committee approved the protocols. Locally bred male Sprague-Dawley rats were obtained at the time of weaning (age 3 weeks). Pre-injection studies were done at age 3 1/2 weeks. Cisterna magna injections were done before 4 weeks age and the animals were studied by magnetic resonance (MR) 1 or 3 weeks later. The rats were then sacrificed immediately following final MR imaging. To induce hydrocephalus, rats were anesthetized with isoflurane (1.5-2% in 100% oxygen). After shaving, cleansing the skin with chlorhexidine and flexing the neck, a 30-gauge needle was inserted into the cisterna magna and 0.025-0.030 ml of sterile kaolin suspension (aluminum silicate; Sigma, St. Louis MO, USA) (250 mg/ml in 0.9% saline) was injected slowly. Sham operated animals were subjected to needle insertion alone. Rats were monitored during recovery from anesthetic, weighed daily, and observed for signs of neurological impairment. They were housed 2 or 3 to a cage and allowed free access to standard pellet food and water. All were sacrificed within 24 h of their final MR imaging. For the MR imaging/water content study a total of 35 rats were used; some rats were imaged both 1 and 3 weeks after injection. For the BBB tracer study 30 rats were used.

### Magnetic resonance imaging and analysis

Proton MR imaging was carried out using a Bruker Biospec/3 MR scanner equipped with a 21 cm bore magnet operating at a field of 7T. The MR probe used was a custom-built quadrature volume coil (length 5.0 cm, inner diameter 3.2 cm) [34]. The rats were anesthetized with 1.5 -2% isoflurane in a 70:30 mixture of N_2_O:O_2_. Their rectal temperature monitored and controlled using a water blanket and cooling air. Respiration was monitored via EKG electrodes placed across the chest and a respiratory monitor. All MR images were taken using magnetization-prepared TurboFLASH sequences with a TR of 3.7 ms, TE of 2.3 ms, FOV of 4.0 × 4.0 cm^2^, slice thickness of 2.0 mm, and matrix size of 128 × 128. The slice taken was a coronal slice 6.8 mm posterior to the division between the olfactory bulb and the forebrain. This level includes the frontal horns of the lateral ventricles and the striatum. Total imaging time for the experiments described below was approximately 1.5 h.

Quantitative diffusion maps were measured using magnetization-prepared TurboFLASH as modified by Thomas and coworkers [35]. It consists of a driven-equilibrium Fourier transform (DEFT) sequence with a pair of diffusion-sensitizing gradients around the 180° refocusing RF pulse followed by the TurboFLASH imaging sequence. Linear phase encoding was used with the central line of k-space shifted to the 25^th ^phase-encoding step to minimize longitudinal relaxation time (T1) recovery during the imaging sequence. Thirty-two averages were accumulated for each image. To quantify the apparent diffusion constant (ADC) of water, a series of images was produced with varying degrees of diffusion weighting (expressed as a *b *value) by increasing the strength of the diffusion gradient. For each *b *value, two images need to be taken differing by a 90° phase shift in the second 90° RF pulse of the DEFT preparation and the imaging pulses. When these two images are combined, images free of eddy current artifacts are obtained. The combination algebra consists of a pixel-by-pixel calculation of the square root of the sum of the square of the individual image intensity. Images with 8 different *b *values of 21, 84, 189, 336, 525, 756, 1029, 1344 s/mm^2 ^were obtained and ADC values were calculated from the decay constant obtained by pixel-by-pixel fitting of the image series to a simple exponential decay.

Maps of T1 were acquired using an inversion-recovery prepared TurboFLASH imaging technique [36]. An adiabatic hyperbolic secant slice-selective pulse with slice-position centered on the imaging slice and slice-thickness of 5 mm was used as inversion pulse. The TurboFLASH readout image was then acquired following a series of effective inversion times of 246, 500, 810, 1189, 1678, 2367, 3546 and 8738 ms. The effective inversion time was calculated as the time elapsed between the inversion pulse and the central line of k-space. Eight signal averages were acquired. T1 parameter images were calculated by fitting the inversion-recovery image series to an exponential recovery curve.

In order to detect water as shown by enhanced transverse relaxation time (T2) values, the spin-echo sequence used for the diffusion experiment described above was used in a different manner. Here, the diffusion gradients were kept small and constant at *b *= 21 s/mm^2 ^and used simply as crusher gradients. The echo times were varied to produce an image intensity dependence on T2. TE's of 40, 60, 80, 100, 120, 140, 160, 400 ms and 48 averages were used. The slice was positioned using a coronal scout image taken 4.5 mm from the top of brain. T2 parameter images were calculated by fitting the image series point-by-point to a simple exponential decay.

**Figure 1:**
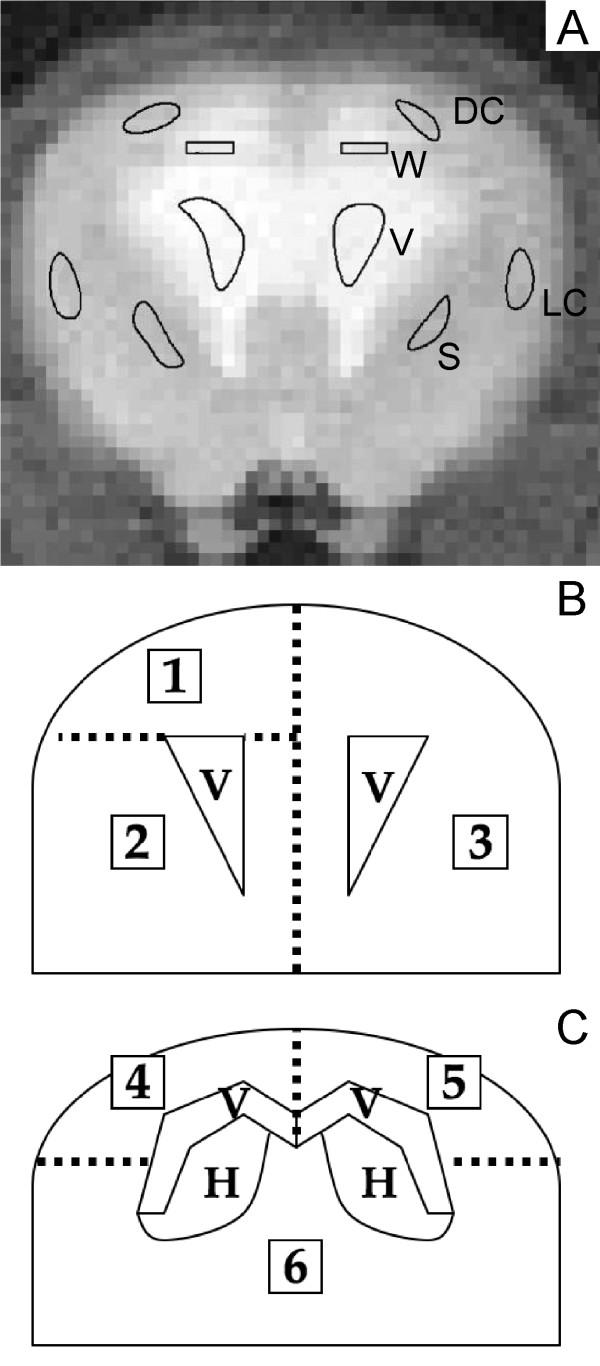
**Brain regions of study**. A: Proton MR image of a coronal slice of rat brain obtained 1 week after kaolin injection into the cisterna magna. The lateral ventricles are enlarged and signal intensity in the white matter is increased due to edema. Representative regions of interest (ROIs) used for determination of MR parameters are shown in the dorsal cerebral cortex (DC), lateral cerebral cortex (LC), striatum (S), periventricular white matter (W), and ventricle (V). B and C: Schematic diagrams of rat brain showing regions sampled for water content and tissue density measurements. The dashed lines represent planes of dissection. The brain was cut in the coronal plane at the level of the optic chiasm approximately in the middle of the slice used for the MR imaging. Anterior (B) and posterior (C) to this plane the slices were divided in the midline. Samples 1 and 4 were used for density measurements. Samples 2, 5, and 6 were used for water content determination by wet-dry weight measurement. The left anterior cerebrum (sample 3) was fixed for histological examination. The dorsal cerebrum samples (1, 4, 5) include cerebral cortex and white matter superior to the roof of the lateral ventricle. Ventral sample 2 includes striatum and lateral cortex. Ventral sample 6 includes lateral cortex, anterior hippocampus, and thalamus. V = ventricle, H = hippocampus.

Regions of interest (ROI) from four anatomical locations (dorsal cerebral cortex, lateral cerebral cortex, striatum, and periventricular white matter) were selected bilaterally (Figure 1A). Different image types (i.e. proton, T1, and T2) were reviewed carefully for all samples to select "pure" tissue samples. The regions had to be drawn separately for each animal due to individual variations in brain and ventricle size. ROIs for all animals were of similar size and were representative of the same anatomical areas. The white matter could not always be clearly distinguished from the ventricle in hydrocephalic rats. Accurate ventricular ROIs could not be assigned for the control animals because the width of the ventricle was less than the width of one pixel (312 μm). The ROIs were saved as a template and overlaid onto the image data sets to calculate values for T1, T2, ADCx and ADCy in each of the regions. Ventriculomegaly was quantified in each of the animals by calculating the area occupied by the ventricles and the area occupied by brain on the T1-weighted image.

### MR imaging and Gd-DTPA administration

**Figure 2:**
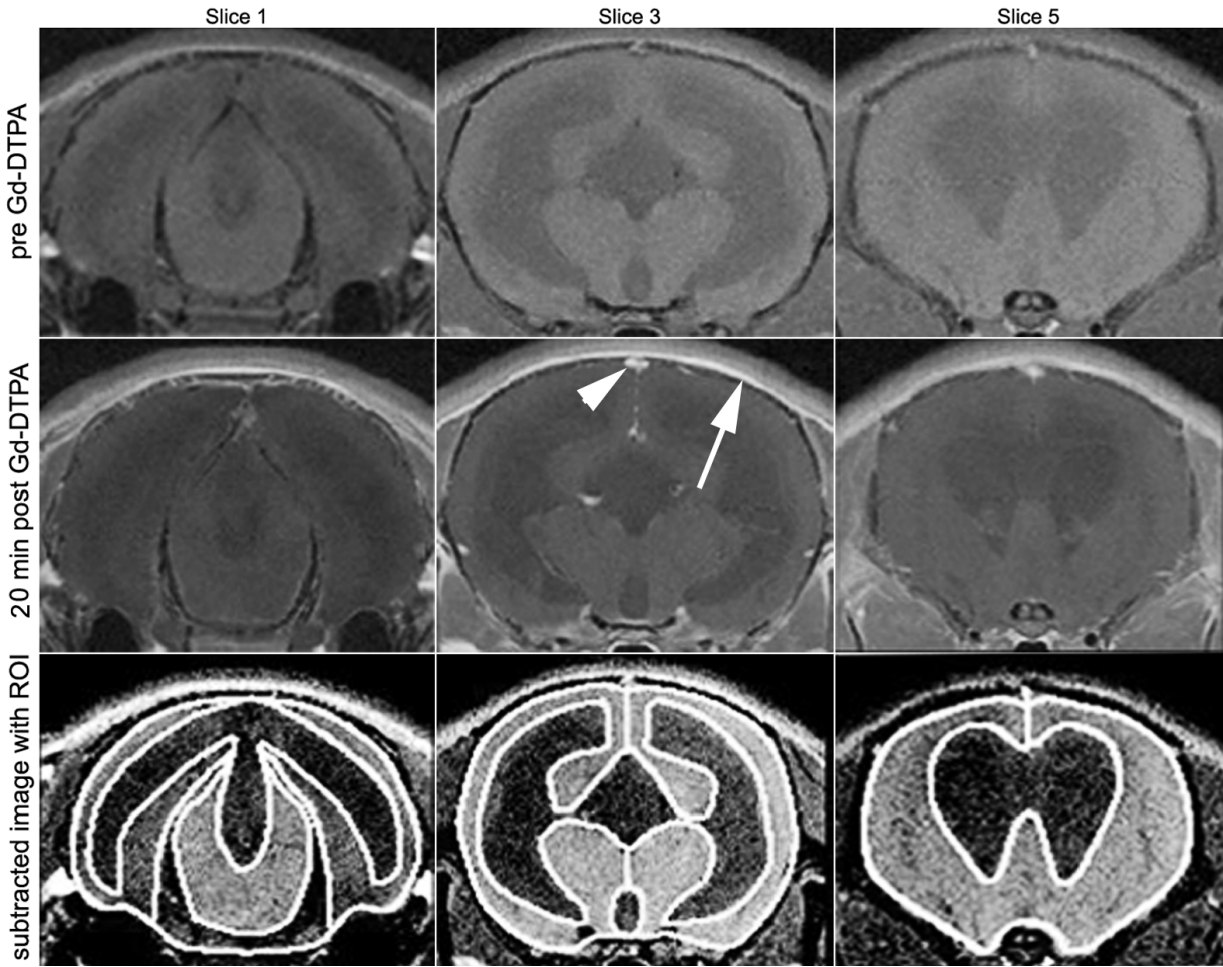
**Subtractive contrast magnetic resonance imaging method**. Coronal MR images (slices 1, 3, and 5) through a rat brain 1 week after kaolin injection demonstrating the subtractive method for quantitative detection of Gd-DTPA accumulation. The upper row shows T1-weighted images obtained prior to Gd-DTPA injection, the middle row shows T1-weighted images obtained 20 min after Gd-DTPA injection. Increased signal is apparent only in the periosteum (arrow) and vascular structures such as the superior sagittal sinus (arrowhead). The bottom row shows the calculated difference images. Note that the entire brain has increased signal due to detection of Gd-DTPA within the vasculature. Areas of extravasation (not evident in this example) would appear brighter. The white lines outline the brain region of interest selected for intensity measurements.

At 1 or 3 weeks post-kaolin injection (10 kaolin and 5 control at each time point), rats were imaged with a volume coil 32 mm diameter and the following parameters: six slices 1 mm thickness with interslice gap of 1 mm; in-plane voxel resolution 0.117 mm × 0.117 mm; recovery time 600 ms; echo time 13 ms. Following anesthesia, a 30-gauge tail vein cannula was placed. A set of pre-contrast T1-weighted images was obtained then Gd-DTPA was injected as a bolus (0.4 mM/kg, volume range 0.1-0.2 mL) and sets of contrast enhanced T1-weighted images were obtained at 10 and 20 min. The ventricle size was calculated by a ratio of the ventricle area to the total brain area on the two slices that include the frontal horns of the lateral ventricles. The percent intensity increase due to contrast enhancement was calculated voxel by voxel using the T1-weighted MR images obtained pre- and 20-min post-Gd-DTPA injection as ((20 min post-contrast image - pre-contrast image)/pre-contrast image) × 100. T1-weighted images were used to draw ROIs omitting the ventricles. ROIs outlining the entire cerebrum and ventricular system were taken from pre-contrast enhanced T1-weighted images (TE = 134 ms) (Marevisi software; National Research Council, Canada). These ROIs were then superimposed onto the calculated percent enhancement images to quantify the average percent intensity increase within the entire brain parenchyma (Figure 2). These methods have been previously described in detail [31].

### Tracer injection and tissue processing

Following MR imaging, a combination of dextran tracers (Texas Red 10 kDa and Fluorescein 500 kDa; Invitrogen Molecular Probes, CA, USA) at a dosage of 5 mg/100 g body weight in sterile saline at a volume of 300-500 μL, was injected into the cardiac left ventricle and allowed to circulate for 60 s, based on previous studies [37]. Rats were then perfusion-fixed with 3% paraformaldehyde in phosphate-buffered saline; brains were removed and divided into three coronal slices by cutting at the levels of the optic chiasm and the mamillary bodies. Brain slices were embedded in paraffin and cut in 6 μM sections. Sections were dewaxed, dehydrated, mounted with Permount and cover-slipped. Representative sections from each level were also stained with hematoxylin and eosin for assessment of morphologic details. Tissues were examined under fluorescence epi-illumination at 40×, 100×, 200× and 400× magnification, by an observer blinded to the duration of hydrocephalus (it is not possible to blind for control vs. hydrocephalic). Regions of tracer leakage were defined when there was clear evidence of fluorescence outside of the vessel lumen. Regions of tracer leakage were plotted onto a diagram of the brain and then were compared to the MR imaging results.

### Immunohistochemistry

To address the hypothesis that caveolin expression might be upregulated in regions of BBB breakdown [38], slides from each brain were immunostained using the following protocol. Sections were pretreated with 0.5% pepsin in 0.01 M HCl for 30 min at 37°C then rinsed, endogenous peroxidases were quenched with 3% hydrogen peroxide in methanol solution for 30 min, sections were blocked with 10% sheep serum in PBS with 0.02% sodium dodecyl sulfate (SDS) for 30 min at room temperature in a humid chamber, the monoclonal anti-caveolin-1 (clone #2297; Becton-Dickenson Biosciences, Mississauga, ON, Canada; diluted 1:15) was applied overnight at 4°C. The secondary biotinylated sheep anti-mouse antibody was applied for 2 h at room temperature followed by streptavidin/HRP for 30 min, then diaminobenzidine. Negative control included omission of primary antibody.

### Water content and density determinations

We used two methods for determining tissue water content. The wet/dry weight comparison, while being the best direct method, is limited to relatively large tissue samples because small samples are subject to rapid evaporative loss from surfaces [39]. Density determination with a gradient column permits the use of smaller samples, for example of cortex and white matter separately [40], however this parameter is not a true measurement of tissue water alone because it is influenced by lipid content [39]. Within 24 h of the final MR imaging, rats were deeply anesthetized and sacrificed by exsanguination. The intact brain was rapidly removed from the skull and dissected over ice-cooled foil. The brain was divided in the coronal plane through the optic chiasm (approximately in the middle of the plane used for MR data acquisition). The anterior and posterior brain pieces were dissected (Figure 1B and 1C). For water content determination, the posterior ventral cerebrum including portions of the anterior hippocampi and thalamus, the anterior ventral cerebrum including lateral cortex and striatum and the left dorsal cerebrum including cortex and periventricular white matter were placed in pre-weighed aluminum foil sheets. These samples were immediately weighed then heated at 100°C for 96 h and weighed again. The percent water content was calculated from the weight loss [39].

For density determination the left anterior dorsal cerebrum and right posterior dorsal cerebrum were placed flat on aluminum foil to preserve orientation of the white matter and immediately submerged in liquid nitrogen. They were stored in sealed containers at -70°C until use. It has been previously shown the density does not change when handled in this manner [41]. Percoll density gradient columns were created in 250 ml graduated cylinders as previously described [42]. Briefly, a dense solution of equal volumes of Percoll (Sigma-Aldrich, Canada) and 0.415 M NaCl (density 1.07 g/ml) was gradually diluted by constant addition of 0.342 M NaCl (1.02 g/ml) and the mixture was continuously transferred to the graduated cylinder. The gradients were calibrated using colored beads with densities of 1.018, 1.033, 1.049 and 1.062 g/ml (Percoll Density Marker Bead Kit; Sigma-Aldrich). The previously frozen samples were allowed to warm slightly and a cylindrical core was obtained using a cooled (-20°C) stainless steel tissue punch (inner diameter 4 mm) attached to a micrometer [1]. The micrometer allowed 1 mm-thick frozen slices to be shaved off the sample. The slices were dropped into the Percoll column and heights were recorded every minute until a steady level was reached (approximately 5 min). The lowest point measured was taken as the true density value. Two or three slices were obtained from all samples. The first and middle slices were solely gray matter and the last slice was predominantly, but not completely, periventricular white matter.

### Statistical analysis

All data are presented as mean ± standard error of the mean. Data were analyzed using JMP 9.0 software (SAS Institute, Inc.; Cary, NC, USA). All data sets exhibited a normal distribution. Changes over time were analyzed using ANOVA with Tukey-Kramer post hoc intergroup comparisons. There were statistically significant differences between the different ages therefore hydrocephalus and control groups of the same age were compared using two-tailed Student's t test. Differences of *p *< 0.05 were considered to be statistically significant. The mean values for cortical density and white matter density did not differ significantly therefore the data are presented as pooled populations. The mean values for frontal cerebrum/striatum water content and ventral posterior cerebrum did not differ significantly therefore the data are presented as pooled populations. These did not influence the statistical comparisons between the control and hydrocephalus groups. Correlation coefficients with Fisher's r to z test were determined between the ventricle size, water content, density, and the quantitative MR parameters. Correlations with *p *< 0.05 were considered to be statistically significant.

## Results

### Induction of hydrocephalus

**Figure 3:**
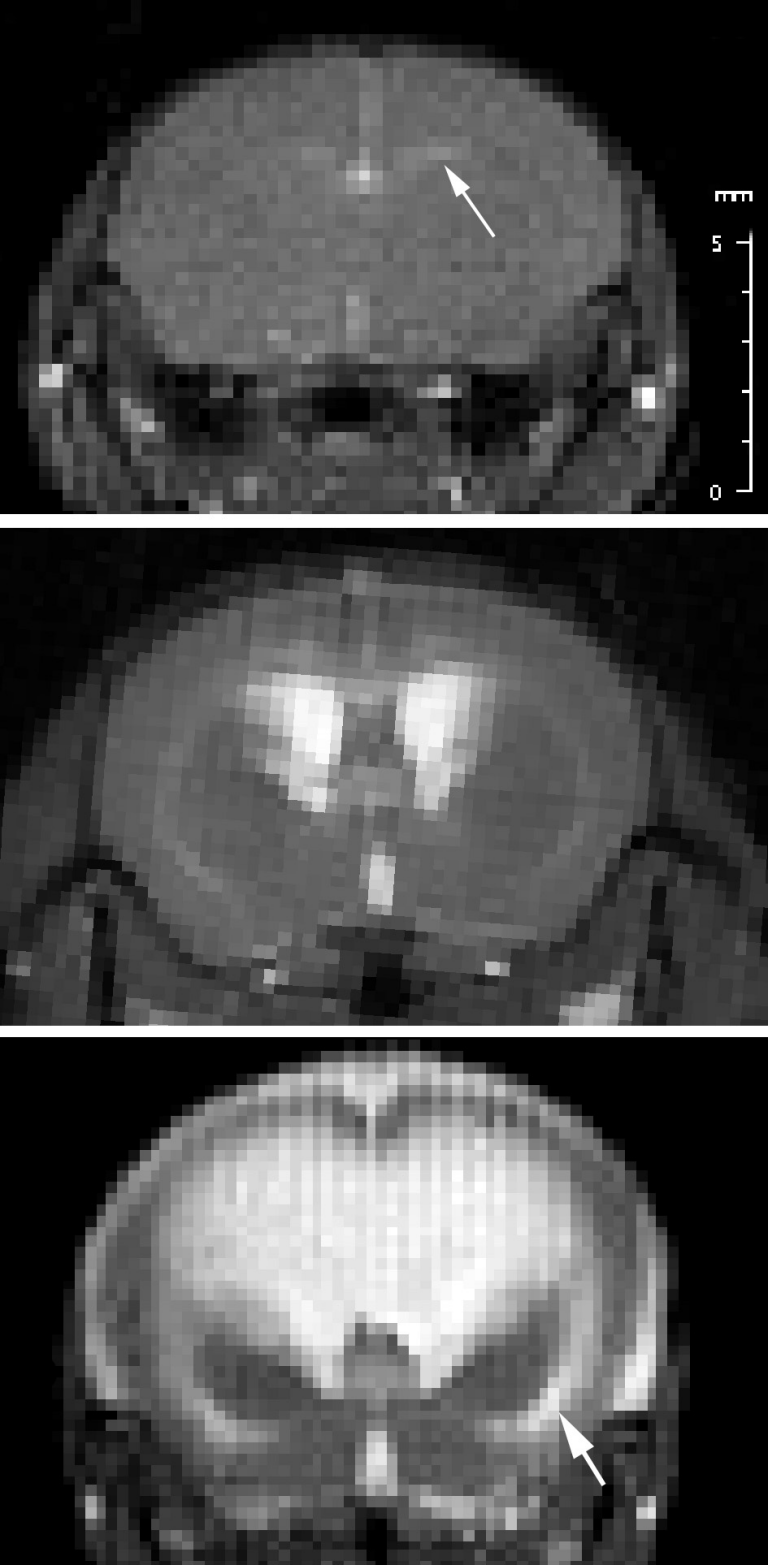
**Magnetic resonance imaging of hydrocephalic rat brain**. T2-weighted magnetic resonance images showing coronal slices through rat brains at the level of the frontal horns of the lateral ventricles, anterior to the third ventricle. Prior to kaolin injection (upper panel) the ventricles are barely visible (arrow). One week after kaolin injection (middle panel) there is noticeable enlargement of the lateral and third ventricles, and 3 weeks after kaolin injection (lower panel) there is marked enlargement of the ventricles as well as the subarachnoid space. Edema is evident in the external capsule (arrow).

All animals tolerated the injection procedures well. Three rats died during or immediately after MR imaging, apparently from anesthetic complications. Four more rats were excluded from the study because they had not developed significant ventricular enlargement. Hydrocephalic rats exhibited a significant lag in weight gain during the period of study (Table 1). The frontal horns of the cerebral ventricles enlarged progressively following kaolin injection (Figure 3; Table 2). Ventricular dilation was variable in hydrocephalic animals, with a calculated brain to ventricle ratio of 0.21 ± 0.02 (range 0.11 to 0.28) in animals at 1-week post injection, and 0.32 ± 0.07 (range 0.13 to 0.60) in animals at 3 weeks post-injection. Control animals had ventricle-to-brain ratios of 0.001 in both groups.

**Table 1:** Animal age, weight and sample size.

GROUP	Pre-injection	Control 1 week	Hydrocephalus 1 week post kaolin	Control 3 weeks	Hydrocephalus 3 weeks post kaolin
Body weight at time of cisterna magna injection (g)	-	84.3 ± 10.2	72.0 ± 7.4	86.0 ± 2.8	80.9 ± 1.6

Age at time of study (weeks)	3.5	5	5	7	7

Body weight at time of study (g)	79.2 ± 6.3 **	131.8 ± 10.5	97.2 ± 6.9 *	262.8 ± 10.6	185.0 ± 13.8 **

Magnetic resonance data: sample size	4	9	12	8	7

Water content/density: sample size	5	4	5	4	8

All values are mean ± standard error. Age groups were compared with ANOVA. Significant differences between the pre-injection group and the 3-week control group are shown in the pre-injection column. Control and hydrocephalus groups of same age were compared with Student's t test. Differences between them are shown in the hydrocephalus columns, * *p *< 0.05, ** *p *< 0.01.

**Table 2:** MR and water content data.

	Pre- injection	Control 1 week	Hydrocephalus 1 week post kaolin	Control 3 weeks	Hydrocephalus 3 weeks post kaolin
Ventricle/brain area ratio	0.001 ± .001	0.002 ± .002	0.063 ± .021 **	0.006 ± .004	0.256 ± .048 **

T1 dorsal cortex	1.83 ± .05	1.88 ± .02	2.07 ± .05 **	1.81 ± .01	2.05 ± .08 **

T1 lateral cortex	1.78 ± .04	1.81 ± .01	1.86 ± .02 *	1.77 ± .01	1.76 ± .03

T1 striatum	1.71 ± .05	1.77 ± .02	1.80 ± .02	1.75 ± .01	1.76 ± .02

T1 white matter	1.72 ± .08	1.75 ± .01	2.87 ± .21 **	1.64 ± .03	2.77 ± .24 **

T2 dorsal cortex	50.5 ± 0.8	49.0 ± 1.9	53.0 ± 2.2	50.5 ± 0.1	55.7 ± 4.4

T2 lateral cortex	48.4 ± 0.3	47.3 ± 2.1	50.3 ± 0.5	49.1 ± 0.3	48.4 ± 1.1

T2 striatum	50.0 ± 0.3	47.8 ± 1.8	50.0 ± 0.4	49.5 ± 0.2	54.8 ± 3.6

T2 white matter	49.2 ± 0.7	49.1 ± 3.5	106.2 ± 11.1 **	49.5 ± 0.6	110.5 ± 17.7 **

ADCx dorsal cortex	716 ± 37	1054 ± 137	1020 ± 152	906 ± 112	1155 ± 132

ADCy dorsal cortex	1238 ± 24	1527 ± 170	1673 ± 109	1704 ± 200	1531 ± 140

ADCx lateral cortex	929 ± 32	1221 ± 151	1450 ± 215	1090 ± 73	930 ± 97

ADCy lateral cortex	896 ± 25	1145 ± 105	1720 ± 207	1239 ± 148	1109 ± 135

ADCx striatum	705 ± 24	992 ± 135	968 ± 126	892 ± 63	706 ± 84

ADCy striatum	1003 ± 20	1297 ± 142	1380 ± 105	1433 ± 184	1233 ± 177

ADCx white matter	1260 ± 67	1578 ± 174	2359 ± 329 *	1436 ± 112	2061 ± 234 *

ADCy white matter	851 ± 27	1158 ± 129	1954 ± 182 **	1253 ± 180	1958 ± 141 **

Density - dorsal cortex	1.034 ± .001 **	1.037 ± .001	1.036 ± .001	1.038 ± .001	1.036 ± .001 **

Density - white matter	1.032 ± .001 *	1.035 ± .001	1.033 ± .001	1.036 ± .001	1.033 ± .001 **

Water content - dorsal parietal cerebrum	81.59 ± .14**	80.52 ± .13	82.36 ± .51**	79.20 ± .20	83.23 ± .49 **

Water content - basal cerebrum	81.16 ± .32 **	79.97 ± .13	80.76 ± .24 *	79.21 ± .21	79.93 ± .19 *

T1 and T2 - longitudinal and transverse relaxation times (msec). ADC - apparent diffusion coefficients (μm^2^/sec). All values are mean ± standard error. Age groups were compared with ANOVA. Significant differences between the pre-injection group and the 3-week control group are shown in the pre-injection column. Control and hydrocephalus groups of same age were compared with Student's t test. Differences between them are shown in the hydrocephalus columns, * *p *< 0.05, ** *p *< 0.01.

### Magnetic resonance parameters

Age of the rats at the time of study, body weight, and sample sizes are shown in Table 1. Quantitative MR values for the ventricle size, T1 and T2, ADC in the × (left-right) and y (dorso-ventral) orientations obtained for the four anatomical regions are shown in Table 2. Among the non-hydrocephalic control rats there was a statistically significant decrease in water content and increase in tissue density as the rats matured from 3 to 7 weeks age. ADCy exhibited a trend to increasing in all locations with maturation. In all locations the T1 and ADCx was highest at 7 weeks age and the T2 was lowest at the same age. As we have documented before [3], cortical ADC values tended to be greater in the direction parallel to the major dendrites and penetrating blood vessels, i.e. in dorsal cortex ADCy > ADCx, and in lateral cortex ADCx > ADCy. Similarly, in the white matter (periventricular and callosal axons) ADCx > ADCy, in parallel with the axons.

The T1 relaxation times in cortex and white matter, but not striatum, were significantly increased 1 week after kaolin injection. By 3 weeks post-injection, despite progressive ventricular enlargement, they had normalized in the lateral cerebrum, but remained elevated in the dorsal cerebrum and in the periventricular white matter. The latter two regions are the ones that exhibit the greatest distortion as the head enlarges. The T2 relaxation times all showed the same trend, however, because the magnitude of change was less and because variability was greater, the differences were not statistically significant, except for the white matter, which was significantly elevated. ADC values were also significantly elevated in white matter, but not in cortex or striatum, at 1 and 3 weeks.

### Water content and density correlations

Table 2 shows the water content and tissue density data. A significant age-related decline in water content and increase in tissue density was observed between 3 and 6 weeks age. Hydrocephalus was associated with increases in water content of the dorsal parietal cerebrum (which includes white matter) and to a lesser degree the basal cerebrum. Data from comparable anatomical sites were used for determination of correlations.

In the dorsal cerebrum, considering controls and hydrocephalics together, ventricle size correlated significantly with water content (combined gray and white matter), cortex density, T1, T2, ADCx, and ADCy (r = 0.617, -0.334, 0.783, 0.471, 0.475, -0.362 respectively, all *p *< 0.03). Considering only the hydrocephalic rats at 1-week, ventricle size correlated significantly with dorsal water content, T1, T2, ADCx, and ADCy (r = 0.952, 0.967, 0.725, 0.896, 0.796 respectively; all *p *< 0.006) but the correlation with tissue density was lost. All of the MR parameters were highly correlated with water content (r value range 0.622 to 0.997, all *p *< 0.03) and with each other. In the 3-week hydrocephalic group, ventricle size correlated only with water content, and ADCy (0.793 and -0.805; *p *< 0.0025).

In the periventricular white matter, considering controls and hydrocephalics together, ventricle size correlated significantly with T1, T2, ADCx, and ADCy (r = 0.685, 0.624, 0.529, 0.510 respectively; all *p *< 0.005), but not with density. Considering only the hydrocephalic rats at 1-week, these correlations were stronger (r = 0.899 to 0.992, all *p *< 0.0001). The correlations were weak in the 3-week hydrocephalic group.

In the basal cerebrum (which for water content analysis includes striatum), considering controls and hydrocephalics together, ventricle size correlated significantly with T2, ADCx, and ADCy (r = 0.722, -0.456, -0.457; *p *< 0.005) but not basal water content or T1. Considering only the hydrocephalic rats at 1-week, ventricle size correlated with water content, T1, and ADCx (r = 0.928, 0.801, -0.760; *p *< 0.03).

### Gadolinium-DTPA permeability

**Figure 4:**
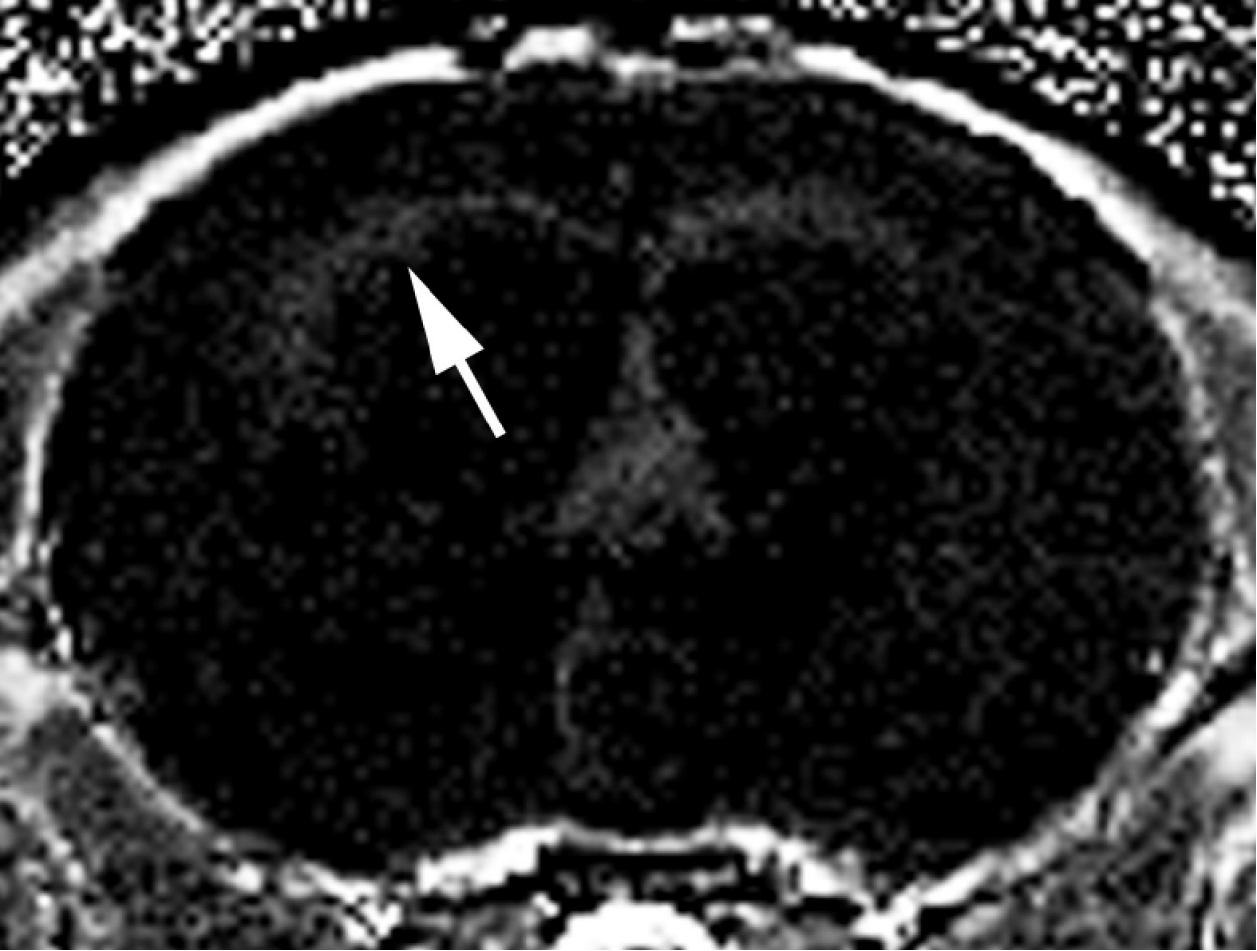
**Magnetic resonance enhancement of periventricular region**. Subtracted difference MR image obtained 20 min after Gd-DTPA injection in a rat that received kaolin injection 1 week earlier. There is a region of signal enhancement surrounding the frontal horns of lateral ventricle (arrow).

**Figure 5:**
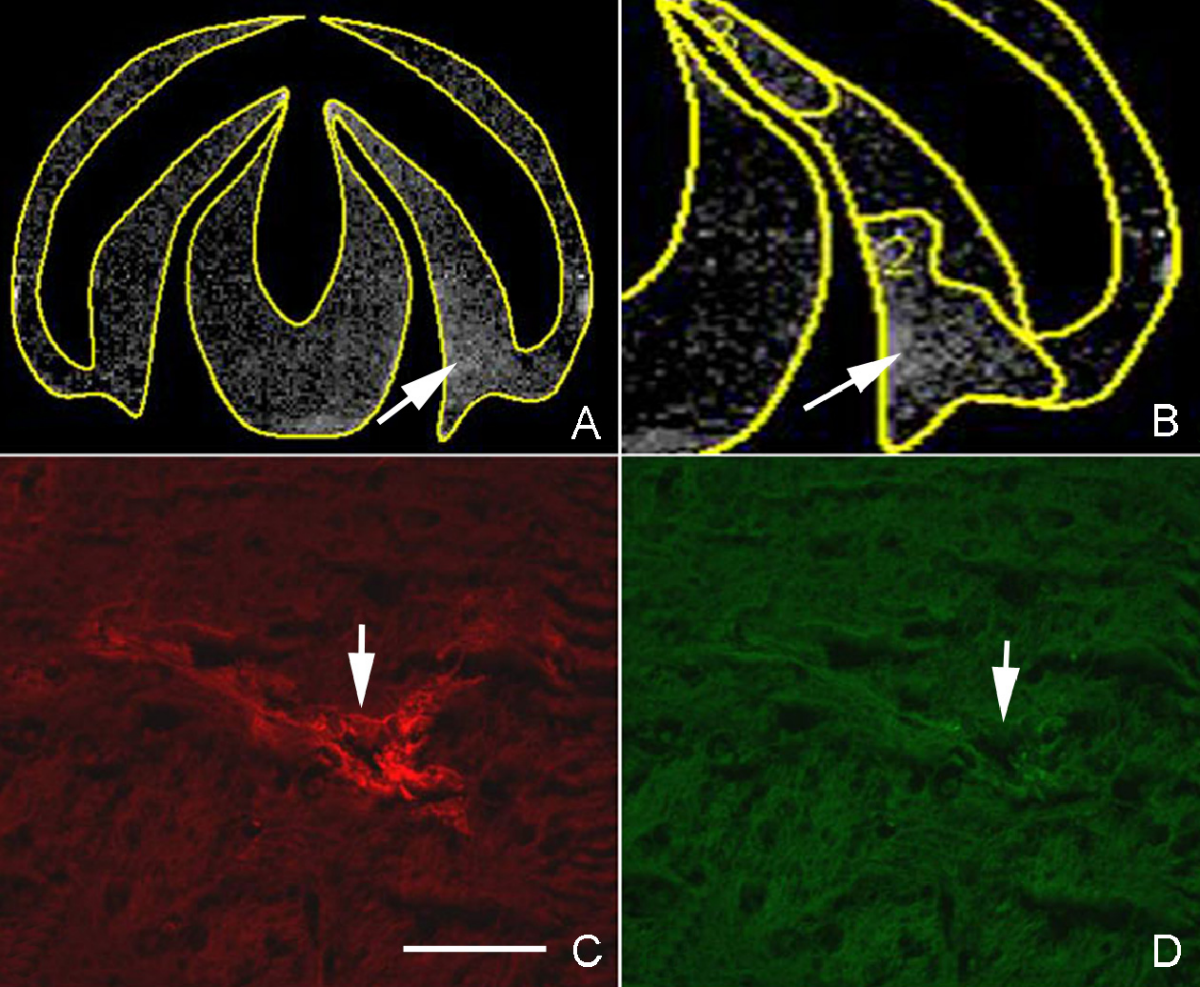
**Magnetic resonance and fluorescent tracer permeability in hydrocephalic rat brain**. MR images and fluorescence microscopy images from a rat that received kaolin injection 1 week earlier. A: Calculated difference MR image obtained 20 min after Gd-DTPA injection through posterior cerebrum and midbrain demonstrating pixels with intensity that increased > 20%. B: Magnification of enhanced signal region in the inferior parietal region (arrow), which suggests that there is BBB disruption with extravasation of Gd-DTPA. C: Photomicrograph of tissue corresponding to signal-enhanced region in B. There is focal diffusion of 10kDa Texas Red tracer into tissue surrounding a vein. D: There is no extravasation of 500kDa Fluorescein green tracer in this area (arrow). Bar = 50 μm.

MR imaging was conducted prior to and again 10 and 20 min following Gd-DTPA injection. Unprocessed images had no obvious areas of signal enhancement. The average pixel intensity in the entire brain was 13% greater after Gd-DTPA in both control and hydrocephalic animals, excluding the area within the ventricles. This likely reflects circulating intravascular Gd-DTPA. On the calculated difference images at 20 min we observed subtle signal enhancement surrounding the frontal horns of the lateral ventricles in 7/20 hydrocephalics (both time points) and 2/10 controls (Figure 4). Using threshold calculations we displayed pixels whose intensity increased > 20% above control value. Control animals did not display any regions of pixel intensity above this value. Among the 20 hydrocephalic rats, 10 displayed irregular asymmetric foci of signal enhancement (7 at 1 week post-injection; 3 at 3 weeks post-injection) (Figure 5a, b). There were no more than 3-4 foci per brain located mainly in the posterior periventricular region, and rarely the posterior thalamus adjacent to the third ventricle or adjacent to the frontal horns of the lateral ventricles. The area of signal change was 1-1.5 mm greatest dimension. There was no obvious difference between 1 and 3 weeks post kaolin injection.

### Fluorescent tracer extravasation

In all brains including controls, blood vessels and choroid plexus exhibited strong red (10 kDa) and green (500 kDa) fluorescence. The ependymal lining of the ventricles also exhibited brighter fluorescence than the surrounding parenchyma, comparable to the Gd-enhancement seen on MR images. There was no evidence of tracer extravasation in any of the control animals. All 20 hydrocephalic animals displayed small foci of 10-kDa tracer extravasation in the frontal and posterior periventricular white matter and the thalamus adjacent to the third ventricle. There was no tracer extravasation in the neocortex, striatum or hippocampus. Of the three coronal slides examined per brain there were typically 0-1 foci of extravasation with the exception of a single slice that had 3 foci. Thin-walled vessels larger than capillaries (probably veins) could be identified near most extravasation sites. There was no evidence of hemorrhage or kaolin-containing macrophages at these sites. Tracer extravasation extended < 50 μm from vessel walls (Figure 5c, d). These regions corresponded relatively well to the regions of Gd-DTPA leakage on the MR analysis. Only 1 hydrocephalic animal (with moderate ventricular dilatation) displayed extravasation of the 500,000 MW tracer at a single site.

### Caveolin-1 expression

**Figure 6:**
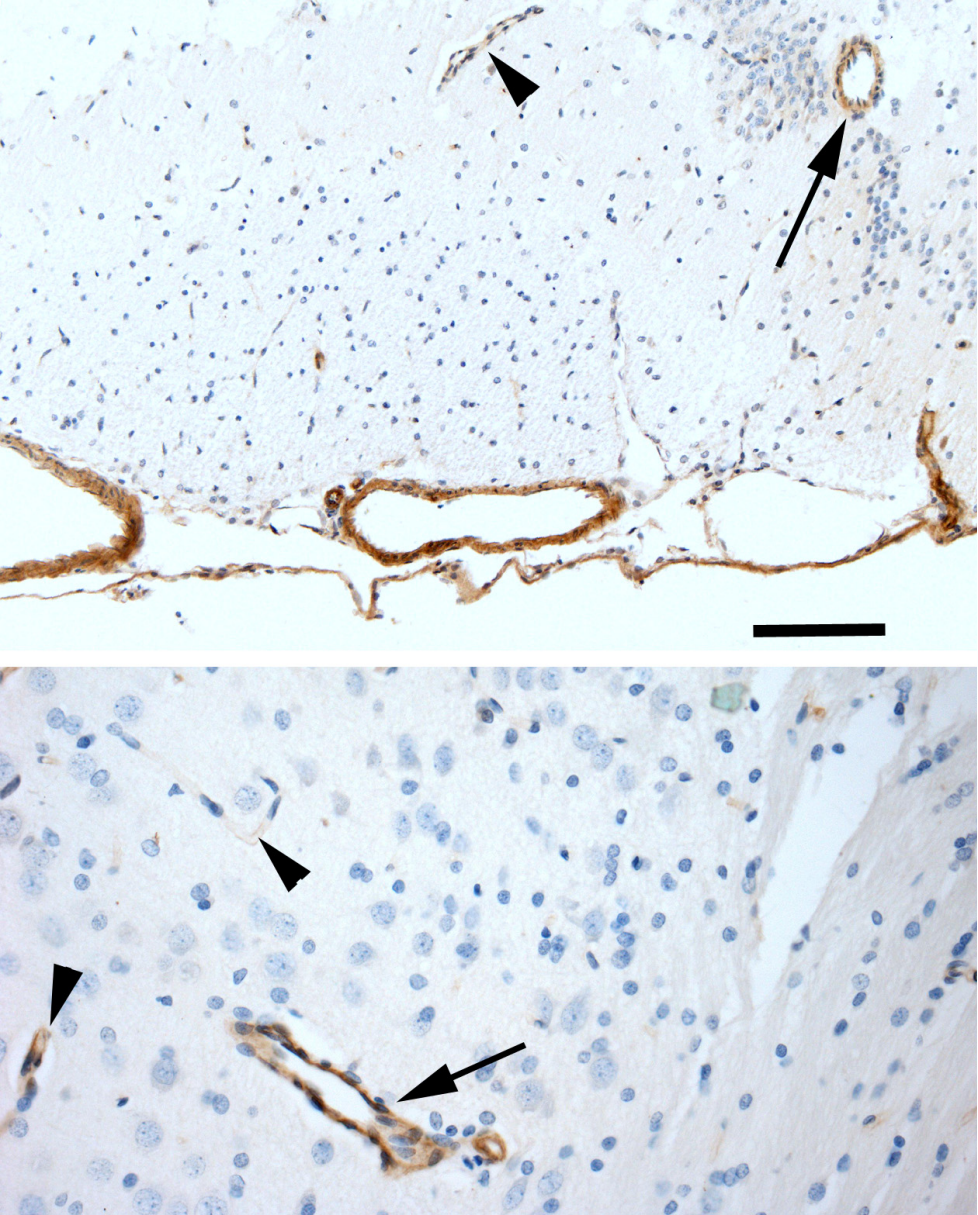
**Caveolin-1 immunoreactivity in hydrocephalic rat brain**. Upper panel shows base of control rat brain with strong immunoreactivity (brown) in surface arteries and arachnoid, as well as in penetrating arteries (arrow) and weaker immunoreactivity in veins (arrowhead). Bottom panel shows periventricular white matter (right) and deep cortex (left) of hydrocephalic rat brain 3 weeks after kaolin injection. Strong immunoreactivity is present in a cortical vein (arrow) and moderate to weak immunoreactivity in capillaries (arrowheads). Bar = 200 μm for top panel and 50 μm for bottom panel.

Caveolin-1 immunoreactivity was observed in the endothelial cells lining all brain surface blood vessels, choroid plexus vessels, and arachnoid of hydrocephalic and control rats; this is consistent with previous reports [38]. There was also immunostaining in the endothelium of scattered parenchymal vessels, most prominent in the larger arteries and veins, and approximately half of the capillaries. There was no qualitative difference between control and hydrocephalic brains in the pattern or intensity of caveolin-1 immunoreactivity (Figure 6).

## Discussion

Tracer extravasation into brain parenchyma can occur through two principal mechanisms: through the endothelial cell (transcellular route) or between the endothelial cells (paracellular route) [43]. The transcellular pathway permits entry by passive diffusion of neutral lipophilic substances < 450 Da. Under normal conditions, the paracellular space is obstructed by tight junctions that prevent passage of substances whose molecular weight is > 180 Da [44]. It has been suggested that these pathways are altered in hydrocephalus [20] but, as noted above, the evidence for BBB disruption in hydrocephalus is scant. We hypothesized that BBB disruption could be demonstrated in a rat model of hydrocephalus using MR imaging of the contrast agent Gd-DTPA (590 Da), a well established marker of BBB disruption [45], and microscopic imaging of fluochrome-conjugated dextran tracers, which have no affinity for specific carrier-mediated transport systems and are not lipophilic. The dextran tracers are useful in determining "pore" size in vessels and in pathological situations where there is the possibility of increased vascular permeability [46]. We demonstrated multiple periventricular foci of Gd-DTPA entry into hydrocephalic brains as well as accumulation along the ventricle lining. The latter might reflect leak through the periventricular organs where there is no BBB. We speculate that the focal tracer leakage is a transient phenomenon; repeated MR imaging in the same animal at separate time points might have been able to prove this. Considering that the MR imaging shows foci of Gd-DTPA extravasation only when subtracted images are used (as opposed to routine images), and the fact that the 10 kDa fluorescent tracer extended only small distances from affected vessels, we conclude that the vessel damage is subtle. We postulate that this is the result of random vascular damage due to mechanical stretching known to occur in experimental hydrocephalus [47,48]. Alternately, proliferating capillaries are known to be transiently permeable as they grow [49]; however the absence of endothelial hypertrophy or hyperplasia makes this a less likely explanation. The thalamic sites of permeation are somewhat surprising because there is very little local distortion in hydrocephalic rats. Perhaps this reflects venous backpressure due to restriction at the major outflow routes, for example on the surface of the distorted brain.

We have no evidence from these tracer studies that the BBB change is generalized, progressive or dependent on the severity of hydrocephalus. In brain injuries with severe disruptions of BBB, increased caveolin-1 immunoreactivity begins at 12 h post-injury and peaks at 2 and 4 days [38]. The absence of such a change in hydrocephalic rats further supports the idea that generalized BBB dysfunction is not a major phenomenon. One group claimed that detection of Tc99-diethylenetriamine penta-acetate DTPA in brain using a gamma camera following intravenous injection of the tracer into hydrocephalic rats was evidence of BBB breakdown [50]; however, only a single animal was used in each group and therefore the data cannot be considered credible. Increased levels of S-100 protein (20 kDa) in blood plasma of children with hydrocephalus were claimed to be indicative of BBB disruption [51]; however, this just as likely represents absorption of released S-100 through conventional CSF absorption routes. Although there is no good evidence for increased BBB permeability in hydrocephalus, a single study showed altered transport across the BBB in aged hydrocephalic rats, with accumulation of amyloid beta peptide [28]. It must be noted that the majority of BBB studies in hydrocephalus focus on young animals; the combined pathology of vascular aging has not been considered adequately. An important consideration of BBB integrity relates to the possible future use of pharmacologic agents for brain protection in hydrocephalus; they must be able to traverse the normal BBB.

Three different methods including wet/dry mass comparison, density gradient column measurement, and MR imaging were used to analyze water content-influenced parameters in separate regions of hydrocephalic rat brains. Previous work has shown that whole brain water in immature rats decreases from approximately 82% at 3 weeks age to 80% at 4 weeks age, and over the following 6 weeks decreases to the adult level of approximately 78% [52]. Our findings in control rats correspond very well to these values. In general the tissue density varied inversely with the water content, as would be expected [53]. Also the tissue density was less in the samples containing white matter than in those that were pure gray matter. This is likely because of the enrichment of lipid in myelin, which accumulates rapidly in rat cerebrum between 3 and 6 weeks age [54,55]. As the water content decreased during brain maturation, T1 and T2 relaxation times and apparent diffusion coefficients (ADCs) changed but were not highly correlated with water content. This is likely because MR properties are also influenced by lipid composition (e.g. myelin) and physical arrangement of tissues (e.g. synaptic contacts and cell processes which affect the tortuosity of the extracellular compartment) [56,57]; all change simultaneously as the brain matures. As previously documented there was directional anisotropy evident in the ADC values [3,58]. In the cerebral cortex there was evidence of less restriction of water diffusion in radial directions, i.e. parallel to penetrating vessels and major dendrites. In the white matter diffusion was less restricted in the direction parallel to the major axonal pathways. Our ADC values and the proportionate degree of anisotropy are slightly higher than those previously reported using a different magnet field strength (4.7 T) [59].

Brain samples from hydrocephalic rats exhibited consistently higher water content than those from control rats, the differences being more pronounced in hydrocephalus of longer duration and in dorsal brain regions that were more distorted (i.e. 3 weeks after kaolin injection the dorsal cerebrum exhibited a 4% difference in water content while the difference in the basal cerebrum was only 0.7%). A similar increase in cerebrum water content is reported in the young H-Tx rat [60]. Good correlations between T1 relaxation and time and brain water content have been reported in models of brain edema [61-63]. However, some experiments suggest that the T2 relaxation time can change when the ratio of bound to unbound water is altered without an absolute change in tissue water content [64]. Following the onset of hydrocephalus most regions showed transient increases in ADC, while in the white matter ADC remained elevated. The extracellular compartment in white matter of hydrocephalic brains is known to be enlarged [12], and experimental infusion of extracellular fluid is also associated with increases in ADC [65]. ADC in human brains with hydrocephalus is higher than in non-hydrocephalic brains and it decreases after shunting [66-68].

Potential shortcomings must be considered. We were unable to sample identical regions for all measurements. In particular the periventricular white matter in rats is very thin (< 400 μm [69]) and therefore sampling uncertainty exists. Absolute water content measurements cannot be made accurately on such small samples [39]. Because of the small size of the rat brain, our density measurements lacked the anatomical precision previously needed to determine differences between gray and white matter of hydrocephalic rabbits [1]. In general the values we obtained in the MR experiments correspond well to previously published values, although we cannot explain why the ADCx and ADCy exhibited different temporal variations. Directionality of ADC in cortex and white matter can be explained by the tissue anisotropy [70]. Finally, there is enormous distortion of the brain in young hydrocephalic animals. This may help explain the lack of direct correlation between water content and MR changes, as has previously been reported in acute cerebral ischemia experiments using adult rodents [33]. They may also explain our failure to replicate observations made in adult rats with hydrocephalus. In that experiment T1, T2, and ADC decreased acutely, suggesting brain compression with expulsion of water [3]. It must be acknowledged that the mechanisms of water movement differ considerably from the mechanism of tracer molecule movements; we cannot state with certainty the extent to which water shifts are dictated by changes in extracellular channel flow versus shifts across blood vessel walls. Given that the state of myelin can affect MR parameters [71], we cannot exclude the possibility that the myelin delay in this model of hydrocephalus [54] causes at least some of the MR signal change in the white matter.

## Conclusions

We conclude that hydrocephalus in young rats is associated with random focal disruptions of the blood brain barrier, but that there is no generalized increase in BBB permeability; we cannot exclude the possibility that some transport functions of the BBB might be altered. The change in white matter water content is therefore most likely explained by altered CSF/extracellular fluid flow and not by altered vascular permeability. No single MR characteristic can be used as a surrogate for water content in the immature rat model of hydrocephalus. Extrapolating to the human situation, it is probably reasonable to follow an individual infant over a short period (e.g. pre and post-shunt) using MR measurements as indicators of brain water or tissue tortuosity, but changes over months would have to be interpreted cautiously because of the influence of developmental and reactive cellular changes.

## Competing interests

The authors declare that they have no competing interests.

## Authors' contributions

MRDB planned the experiments and unified the manuscript. IS did the animal models and microscopy. AES and RJB did the subtractive MR imaging and quantitative analysis. TLK performed the water content and density measurements. All authors have read and approved the final version of the manuscript.
